# Views and experiences of young people, their parents/carers and healthcare professionals of the advance care planning process: A summary of the findings from a qualitative study

**DOI:** 10.1177/02692163221083447

**Published:** 2022-03-31

**Authors:** Ben Hughes, Mary O’Brien, Anita Flynn, Katherine Knighting

**Affiliations:** 1 University of Bolton, Bolton, UK; 2 Edge Hill University, Ormskirk, Lancashire, UK

**Keywords:** Qualitative research, advance care planning, young people, adolescent, healthcare personnel

## Abstract

**Background:**

Advance care planning for young people is relatively new in the UK. There is a lack of understanding about the engagement of young people in their own planning process, optimal timing of discussions and the facilitators and barriers to the engagement of young people.

**Aim:**

To explore the views and experiences of young people, their parents/carers and HCPs of the advance care planning process.

**Design:**

A qualitative study, using semi-structured interviews with young people, their parents/carers and healthcare professionals across four case series. Data were analysed using thematic analysis.

**Participants:**

Fifteen participants were interviewed: young people (*n *= 2), parents/carers (*n *= 5) and healthcare professionals (*n *= 8).

**Results:**

Three themes were identified from the findings. Key findings related to barriers and facilitators of engaging young people in their own care planning were apparent in the following areas: misperception of terms; hierarchies of power in relationships; and a flexible and innovative organisational structure and culture.

**Conclusion:**

Participants expressed a variety of views and experiences of advance care planning. Advance care planning was thought to be best initiated by a consultant when the young person is in their mid-teens, their condition is stable, and before they transition to adult care. Engagement was also considered to be facilitated by appropriate communication, developing relationships prior to initiating advance care planning, and written support for everyone involved in the process. These factors were supported by training and education for healthcare professionals and a flexible and innovative structure and cultures of organisations.

**What is already known about the topic?**

- The aim of advance care planning is to include young people in their own planning process where possible.
- Engaging young people in their own advance care planning process is likely to enrich the standard of care they receive.
- Communication, relationships and the training of healthcare professionals can be either a barrier or facilitator to the engagement of young people in the advance care planning process.

**What this paper adds?**

- The optimal timing to initiate advance care planning is by the time a young person is in their mid-teenage years, when their condition is stable, and well before they transition to adult care.
- The main barriers to engaging young people in their own care planning included the misperception of advance care planning; poor levels of health literacy; limited access to education and training for professionals; perceptions of hierarchical relationships; and a rigid organisational structure.
- The main facilitators to engaging young people in their own care planning included clarity of communication; improved health literacy levels; better access to education and training for professionals; and a flexible and innovative organisational structure and culture.

**Implications for practice, theory or policy**

- The engagement of young people and their family in advance care planning can be more accessible with open, honest, sensitive and empathetic communication, reduced use of medicalised language, and a greater focus on health literacy to ensure shared understanding of terms used and choices.
- Young people would benefit from developing trusted relationships prior to initiating advance care planning to reduce miscommunication, misperception of the process and decisional conflict by reducing perceptions of a hierarchy of power between patients and professionals.
- High quality advance care planning requires a flexible and innovative organisational structure and culture which promotes person-centred models of care and invests in affordable and accessible education and training for professionals to develop their confidence and skills to support engagement of young people.

## Introduction

Advance care planning is a voluntary process to involve patients in their own care by sharing their personal values and goals for future care in the event of becoming seriously ill.^1,2^ Discussing and documenting wishes is associated with decreased emergency admissions, reduced hospitalisation and fewer complex treatments and hospital deaths.^3–5^ Advance care planning also helps prepare patients and relatives for death by involving them in the decision-making process.^3,6,7^

Advance care planning for adults is widely practised in the United States, Canada, Australia and New Zealand and is based on preserving personal autonomy in decision-making.^3,8^ A range of policies and aims both within and between organisations and countries has been complicated by different legal frameworks and terminology for advance care planning.^9^ This variety has produced an ambiguous national and international legal framework for advance care planning, which in turn has impeded its implementation.^10^

Advance care planning for adults is well embedded in the United Kingdom, being one element within the Gold Standards Framework which aims to foster high-quality end of life care.^11,12^ Policies and procedures for advance care planning with young people are less developed than for adults,^13^ despite it being identified as a key contributor to effective communication about their care.^14^ The Convention of Children’s Rights (1989) recognises the right for children to be involved in medical decision making. Legislation and policy in the United Kingdom (such as The Children Act 2004 and the Mental Capacity Act 2005) allowed decisions to be made about children according to their best interests and recognised young people’s involvement in their own care decisions.^15–18^ Accordingly, advance care planning for young people exists in United Kingdom policy and its use has increased since 2010.^12,19–21^ However, the limited uptake of young people making an advance care plan reflects the international perspective.^5^

Various documents, including The Wishes Document^22^ and My Choices,^23,24^ have been used for recording advance care planning for young people. Strategies, such as Family Centered Advance Care Planning.^25–29^ have helped engage people in the process. The United Kingdom’s status as one of the leading countries for paediatric palliative care is reflected in the provision of relevant guidelines and resources for advance care planning with young people.^30^ Organisations such as Together for Short Lives and The Council for Disabled Children have developed various resources to guide young people’s care planning and support the use of planning tools.^31,32^ More recently, the Child and Young Person’s Advance Care Plan documentation has been devised in the UK and its use is now spreading throughout the United Kingdom.^33^

Engaging young people in their own planning process can have a positive impact on their anxiety.^24,34^ Professionals felt communication is a key aspect of facilitating this engagement.^35^ Previous research has indicated that good communication is central to advance care planning^36^ and is strengthened by exploring the experiences of the young person.^37^ However, both communication and training for professionals implementing and using advance care planning may be a concern.^36,38,39^ While structured communication may be useful, there is still limited information about optimal timing of advance care planning discussions.^40^ Similarly, resources and the time to use them effectively, have been identified as further potential barriers to using advance care plans in paediatric care.^30,36,38,41^

Over 86,000, and up to an estimated 99,000, children and young people with a life-limiting or life-threatening condition in the United Kingdom may benefit from advance care planning.^42^ Definitions of a ‘young person’ are diverse, ranging from aged 10 to 24 years old ^43^; aged 15 to 24 years old ^44^; under 18 years old ^45,46^; and aged 14 to 18 years to reflect the age of criminal responsibility, and the maturity and capability for independence as people approach adulthood.^47,48^ A ‘young person’ was defined as 13–24 years old for this study to correspond with the Medical Subject Headings definition of a ‘young adult’ and the existing age range used by many children’s hospices.^12,49^

There is a lack of evidence on the barriers and facilitators to engagement, with views and experiences of young people rarely included in research to identify their engagement in their own care planning.^12^ Understanding the experiences and engagement of young people in their care planning process can support the planning and delivery of palliative care because of the increasing life expectancy of young people.^22,50,51^ There is also a lack of evidence on the concurrent experiences of young people, parents/carers and professionals in the process.^12^ This paper presents findings from a qualitative study to understand the views and experiences of all involved and identifies barriers and facilitators to engaging young people in their advance care planning.

## Research question and objectives

The research question was: ‘What are the views and experiences of young people, their parents/carers, and healthcare professionals of the advance care planning process?’ The objectives were to explore the views and experiences of young people, their parents/carers and healthcare professionals on:

1. the use of advance care planning;
2. the timing of the implementation of advance care planning; and
3. the barriers and facilitators to the engagement of young people in the advance care planning process.

## Research design

Multiple case study methodology was used to explore advance care planning for young people from the perspectives of young people, their parent/carers and the professionals involved.^52–54^ Four case studies facilitated within- and cross-comparison of the phenomenon to incorporate different contexts and multiple perspectives.^54,55^

## Definition of the case

The case was defined as a young person aged 13–24 years old, with a life-limiting condition and an advance care plan in place. Case studies were centred around the young person and included a parent/carer and at least one healthcare professional involved in their advance care planning.

## Sampling and participant recruitment

Purposive sampling helped identify young people as the unit of analysis for each case, then to identify each member of the case study.^56^ A nominated member of staff from the clinical team at each site screened their patient list and identified potential ‘cases’ using the inclusion and exclusion criteria (Table 1). Once potential cases had been identified, recruitment was split into two stages to build the case series.

**Table 1.** Inclusion and exclusion criteria for the recruitment of young people.

Inclusion criteria	Exclusion criteria
The young person will be: 1. Aged 13–24 years 2. Have a life-limiting condition 3. Have an advance care plan in place 4. Identified by a clinical lead at the named clinical sites 5. Although not a criterion to be a case for the study, the young person will have relatively intact verbal communication or other established method of communication to participate in the data collection phase of the study themselves	The young person will be excluded if they do not meet all of the inclusion criteria or: 1. They are too unwell to be interviewed 2. The young person is considered imminently close to death as indicated by the clinical team 3. Parents/carers do not give their consent for the young person to be included in the interview phase of the study 4. The young person does not give consent/assent to be interviewed

### Stage one: The recruitment of young people and their parents/carers

In line with advice from the National Health Service Research Ethics Committee, potential participants under 16 years of age were contacted through the parents/carers. Those aged 16 years and above were contacted at the same time as their parents/carers. Inclusion and exclusion criteria for the recruitment of parents/carers are outlined in Table 2. The nominated staff member gave the young person or their parent/carer a flyer about the research, enabling them to contact the researcher (BH) to express an interest in the study and ask questions. At this point, eligibility to participate was checked and the participant information sheet and consent form were sent to potential participants to introduce the lead researcher and outline the aims of the study.

**Table 2.** Inclusion and exclusion criteria for the recruitment of parents/carers.

Inclusion criteria	Exclusion criteria
1. An advance care plan must be in place for their ‘young person’ who is aged 13–24 years 2. Identified by a clinical lead at the named clinical sites 3. Aged 18+ years old 4. Must be able to communicate fluently in English	1. Their ‘young person’ does not currently use an advance care plan 2. Aged under 18 3. Cannot communicate fluently in English 4. Their ‘young person’ has died before data collection has begun for the parents/carers

### Stage two: The recruitment of healthcare professionals

Healthcare professionals involved in the advance care planning of the ‘case’ were identified by the nominated staff once the young person and/or parent/carer had agreed to participate. Professionals were sent an invitation to participate with the information sheet. During data collection any additional healthcare professionals mentioned were invited to participate if they met the sampling criteria (Table 3).

**Table 3.** Inclusion and exclusion criteria for the recruitment of healthcare professionals.

Inclusion criteria	Exclusion criteria
1. Must be involved in the implementation and use of advance care plans with a young person identified as a ‘case’ in Stage One 2. Working at any professional grade, for the NHS or other healthcare service including children’s hospices 3. Aged 18+ years old 4. Must be able to communicate fluently in English	1. They are not involved with advance care plans for the young people in Stage One of the study

Twenty-four young people met the criteria and were contacted to participate in the study: Nineteen did not respond to the invitation to take part and one withdrew due to poor health. Four cases proceeded to the interview phase of the study.

## Data collection

Informed consent was gathered from all participants before interviews commenced. Written consent was gained at face-to-face interviews; verbal consent was recorded at the beginning of telephone interviews. Semi-structured interviews provided flexibility and opportunities to clarify responses, ask follow-up questions, and identify emerging themes^57–59^ and were used in similar previous studies.^13,60,61^ Seven individuals (young people *n *= 4; parents *n *= 3) were consulted to help shape the language and layout of the research materials and ensure they were accessible for potential participants. As an experienced researcher, BH’s PhD Director of Studies (KK) was present for three initial interviews to help guide and advise the process. Interviews were digitally audio-recorded, transcribed and anonymised by BH. Transcripts were not returned to participants and repeat interviews were not undertaken due to the sensitive nature of discussions and potential deterioration in condition of the young people.^62^ Field notes were made by BH after each interview and a reflective diary was maintained to add transparency to the interpretivist, qualitative research process.^63^

## Data analysis

NVivo 12^©64^ was used as a data management tool to facilitate within- and cross-case analysis. Thematic analysis was used to help recognise patterns in the data,^65,66^ maintain consistency of findings^67^ and identify new themes as data was collected and analysed in a systematic and rigorous way.^65,68–70^ Attride-Sterling’s model of thematic analysis was adopted because it allowed for analytical generalisations within the study’s theoretical framework and so aided the case study methodology. The stages of thematic analysis can be summarised by the following steps: code the data; identify themes based on the text segments and then refine the themes; construct thematic networks (connecting themes using web-like illustrations); describe and explore the thematic networks; summarise the thematic networks; and interpret patterns.^65^ Initial basic, organising and global themes identified by BH were reviewed and discussed with the team (KK, MoB, AF) to enable consensus to be reached on the final themes.

## Permissions and ethical approvals

Institutional ethical approval was obtained (Ref: FOSH145) along with National Health Service Research Ethics Committee approval and Research Authority approval (REC reference: 16/NW/0643; IRAS project ID: 206015).

## Findings

**Figure 1.**
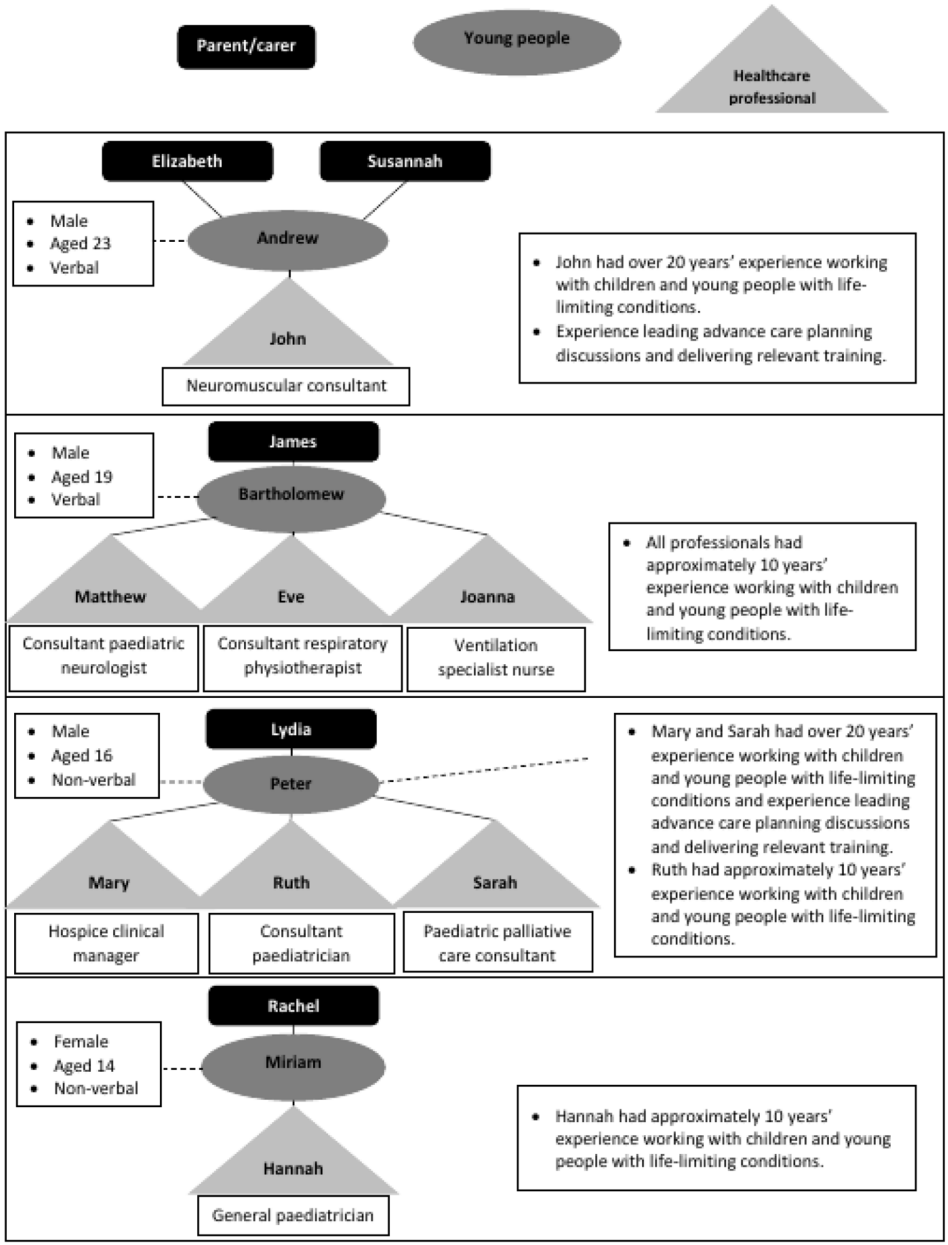
Case studies and participants.

Fifteen participants contributed to four case studies. Interviews were conducted where participants felt most comfortable: clinical setting (*n *= 5), place of work (*n *= 4), home (*n *= 2) and via the telephone (*n *= 4). Interviews were conducted by BH between July 2016 and June 2018. The interviews lasted approximately 20 min with young people and an average of 45 min with parents/carers and healthcare professionals (range of 15–100 min). A data category was considered to be saturated if it was reflected in more than 70% of the interviews.^71^ The case studies and participants are presented in Figure 1. Participants comprised four young people (three male, one female), five parent/carers (one male, four female) and eight healthcare professionals (two males, six females; five consultants, one ventilation nurse specialist, one consultant respiratory physiotherapist, and one hospice clinical manger). All participants were assigned pseudonyms to protect their identity.

The average age of the young people was 18 years, with a range of 14–23 years. Two young people (Andrew and Bartholomew) could communicate verbally, although both had some cognitive impairment which meant they were not always able to express their thoughts clearly. The other two young people (Peter and Miriam) had a range of complex health problems. Consequently, Peter was non-verbal and Miriam was non-communicative, meaning neither could be interviewed for the study. All young people in the study were living at home; three of them were in education and all four were under the care of hospital consultants, with Peter and Miriam also using a hospice. Additional support was provided by organisations such as a centre for adults with neuromuscular conditions and individuals like physiotherapists. Day-to-day care was primarily provided by parents/carers, with further support from paid carers for Andrew and Peter.

To meet the objectives of the study six themes were constructed from the global theme of advance care planning for young people.

### Theme 1: Understanding of advance care planning

Participants felt that in order for advance care planning to be a proactive approach to planning future care, it should reflect the personal views and wishes of the young person, or their parents if the young person was unable to participate in the process:

‘[Advance care planning should] *detail the individuals’* *wishes as they move through their care journey*’ (Eve, Consultant Respiratory Physiotherapist, Bartholomew’s case study).

However, the study revealed misperceptions of advance care planning among some participants. One person confused the process with a carers’ assessment and felt it ensured support was in place for her and her husband:

*‘I suppose I was doing everything and I wasn’t getting any younger. . .we* [Elizabeth and her husband] *really needed care’* (Elizabeth, Grandparent, Andrew’s case study).

Similarly, another parent believed advance care planning would ensure her daughter had a pain-free death:

*‘They [doctors] can bring drugs in to help them [young people] so they’re not in pain and they’ll pass nice instead of all stressed out and everything [sic]’* (Rachel, Parent, Miriam’s case study).

Misperceptions like this impacted on views of care, leading one grandparent to explain she would only allow Andrew (Young Person) to participate if she thought it was appropriate.

*‘I wouldn’t involve him* [Andrew]. *I’d like to find out what it’s all about first’* (Elizabeth, Grandparent, Andrew’s case study).

Such views may have been gatekeeping due to the cognitive ability and deteriorating health of the young people. It was apparent during his interview that Andrew knew what he wanted to say but, like Bartholomew (Young Person), he had difficulty verbalising his thoughts.

There was agreement both within and between case studies that advance care planning should not be a legally binding, rigid document. A flexible approach would allow it to follow young people to different places of care and be understood by relevant professionals through the deterioration of their condition. Importantly, it could also be understood by non-professionals and be more readily updated:

*‘It’s a flexible document, a living document’* (Mary, Hospice Clinical Manager, Peter’s case study).

Participants felt a simple and straightforward process would help involve relevant professionals and engage young people. Simplicity was important because it became apparent through interviews with young people that their cognitive ability impacted on their understanding of information and expression of wishes. An increasing number of responses in the young people interviews, particularly from Bartholomew, were met with a ‘*Don’t know’* response, highlighting the challenge of ensuring young people fully understand the process and are able to engage in a meaningful way to express their wishes.

### Theme 2: Advance care planning for young people in practice

Experiences of advance care planning were mixed across the case studies. The general consensus was that advance care planning should be initiated when young people are in their mid-teens and their condition is stable.

‘[Discussions] *should start before* [the young person becomes really poorly]’ (Mary, Hospice Clinical Manager, Peter’s case study).

Young people also agreed the optimal timing of initiating discussions was prior to transition to adult care:

‘[Discussions should begin] *when you’re going from a child to an adult’* (Andrew, Young Person).

In practice, timing of advance care planning did not always happen like this. In the case studies of Peter and Miriam, the combination of their communicative abilities, cognitive decline, increased hospitalisation and invasive treatment led to advance care plans being discussed, documented and revised at key points instead:

‘[Advance care planning discussions often follow] *more frequent hospital admissions*, [or if the young person is] *needing extra respiratory support from his symptom point of view. Are those really getting out of control now or are you struggling to control those symptoms*, [deterioration in] *quality of life, sleeping more* [or] *less awake time?’* (Ruth, Consultant Paediatrician, Peter’s case study).

There was consensus among all participants that advance care planning should be led by a consultant with a long-standing relationship with the young person and their family because these professionals can provide the medical prognosis around which wishes and plans can be made:

*‘You’ve got more confidence with them’* (Andrew, Young Person).

Thirteen of the fifteen participants also believed young people should be involved in their own care planning decisions if they were able to and wanted to be involved:

*‘I would always say, if they’ve shown an interest, at whatever age, then they should at least have an opportunity to be involved’* (Joanna, Ventilation Specialist Nurse, Bartholomew’s case study).

The remaining two participants felt involving young people in their care decisions should not happen. Elizabeth (Grandparent) felt protective towards allowing Andrew (Young Person) to participate, while a parent believed this approach was cruel:

*‘I’m not talking about that in front of her* [Miriam]. . .*You don’t tell the dog you’re taking them to the vets, do you? . . . That’s how I see it. You wouldn’t tell a dog you’re going to the vets today* [and] *you won’t be coming home. You don’t do stuff like that’* (Rachel, Parent, Miriam’s case study).

Providing appropriate support for young people and their families was identified as a key element to facilitate the engagement of young people and ensure their understanding of the process. Although written information was often available for healthcare professionals, young people and their parents/carers reported a negative experience of advance care planning because they felt support did not filter down to them.

*‘It’s really confusing, frustrating, and scary’* (James, Parent, Bartholomew’s case study).

The case studies also illustrated differences in the documentation of advance care plans. Documenting discussions was considered important to clearly communicate wishes to all who may need to know the information. The Child and Young Person’s Advance Care Plan document was used to record discussions in the case studies of Bartholomew, Peter and Miriam. However, different documentation was used in Andrew’s case study:

*‘Over and over again people say why do we need sixteen pages. . .[A] big criticism is the last page. . .One family described it as an insult. . .the sixty, seventy page one [document], is not an optimal document’* (John, Consultant Paediatric Neurologist, Andrew’s case study).

These criticisms led John to record wishes under different relevant sub-headings in a straightforward word-processed document. This simple method listed different organisations the care plan was distributed to and included signatures of the individuals involved in discussions. Every comment and revision was recorded and agreed by those involved in discussions. However, participants in the study generally felt standardised documentation was beneficial to support consistent practice across settings and share information in a meaningful way with all those involved in the care of the young person:

*‘It made no sense for us to use our own* [document] *because* [it] *needs to be recognised wherever the child goes’* (Mary, Hospice Clinical Manager, Peter’s case study).

In Miriam’s case study, both Rachel (Parent) and Hannah (General Paediatrician) believed they would not expect to see any changes in Miriam’s (Young Person) care and treatment from documenting advance care planning discussions. Hannah (General Paediatrician) felt recording wishes could make carers a bit nervous, although she attributed this anxiety to a misunderstanding of advance care planning and a perception that a deterioration in condition should result in hospitalisation.

### Theme 3: Communication

The variety of healthcare professionals involved in advance care planning necessitates effective communication if the document is to be read and used by all involved in a young person’s care. Although Andrew’s advance care plan was sent to 15 different professionals, the hospice itself did not have a record of the document. Miriam’s case study also revealed instances of distrust about the sharing of information between professionals involved in Miriam’s care during advance care planning discussions:

*‘You say one thing to one nurse and it’s round the bloody building’* (Rachel, Parent, Miriam’s case study).

This highlighted the importance of professionals working with families to develop an open and honest relationship in which sensitive feelings and consideration of future treatment options can be shared and discussed. This approach was believed to foster relationships and provided reassurance for the family:

*‘. . .communication improves, it’s such an intimate* [time for] *discussions and you meet so frequently, families take great reassurance’* (John, Consultant Paediatric Neurologist, Andrew’s case study).

Young people and families require clear and understandable communication about the process and throughout the discussion, but this can be complicated when young people have cognitive impairment or are non-verbal or non-communicative. In Peter’s case study, communication was an evolving process which was dependent on the close, loving relationship between Lydia and her non-verbal son:

‘Being *his mum for sixteen years, his parents for sixteen years, we just know his personality, we know his outlook on life, we know what he likes doing. I’ve got an understanding, which I think would be the same with any parent and child. It’s just a feeling’* (Lydia, Parent, Peter’s case study).

Conversely, emerging conversations in interviews suggested some participants perceived the language and terminology as creating a barrier to engagement for non-clinically trained people or those with cognitive impairment. In Peter’s case study, Lydia (Parent) reported that she and Peter felt insecure because discussions were sometimes beyond their understanding due to these barriers:

‘*I don’t think he* [Peter] *understands the complex language* [used in decision-making as part of his advance care planning]’ (Lydia, Parent, Peter’s case study).

The use of medical language in Peter’s advance care planning process meant Lydia felt insecure because she perceived a hierarchy in communication and discussions, meaning information was sometimes beyond their understanding. This was recognised in Andrew’s case study when John (Consultant Paediatric Neurologist) spoke about the challenge of using appropriate communication with young people and their parents/carers:

‘[Advance care planning with young people needs] *a kind of reflection on language’* (John, Consultant Paediatric Neurologist, Andrew’s case study).

Communication was further complicated by issues around transition to adult services. Andrew’s transition entailed leaving the care of a children’s hospice and establishing communication with a new team. In Bartholomew’s case study, James (Parent) and Matthew (Consultant Paediatric Neurologist) said the future was uncertain because of challenges and uncertainties of transition and the lack of adult services compared to paediatric services:

*‘We don’t [know] about the future plans or we don’t know what to do. . .if [something] goes wrong’* (James, Parent, Bartholomew’s case study).

Overall, participants felt individualised communication, including clarity of information and non-medicalised language, facilitated the understanding and engagement of young people in their care planning.

### Theme 4: Education and training for healthcare professionals

Healthcare professionals had different views and experiences of training. Five of the eight professionals felt training was adequate and met their needs. Most of these participants were experienced in their role and frequently led advance care planning discussions:

*‘I’ve been working in this field for many, many years* [and] *I know this patient group inside out’* (John, Consultant Paediatric Neurologist, Andrew’s case study).

However, other healthcare professionals reported a lack of availability or described barriers to accessing training courses. These participants were often the least experienced in their role or with advance care planning. One exception was in Bartholomew’s case study, where Eve was experienced working with young people with life-limiting conditions and complex healthcare needs but did not feel confident about advance care planning:

*‘Absolutely not, no, no*. [I would like to know] *where to go to get the information or to access the training’* (Eve, Consultant Respiratory Physiotherapist, Bartholomew’s case study).

The absence of accessible training opportunities meant some professionals lacked confidence and doubted their competence to initiate and lead advance care planning discussions, which created a barrier to engaging young people in the care planning process. Two professionals from Peter’s case study worked for a charity and felt that training was too expensive and delivered too far away from their location, adding both a financial and time burden to an already strained budget and workload:

‘[Training] *needs to be more easily available [and is] quite an extortionate cost’* (Mary, Hospice Clinical Manager, Peter’s case study).

This view contrasted with professionals who were more experienced or based in larger, more centralised locations. John (Consultant Paediatric Neurologist, Andrew’s case study), Sarah (Paediatric Palliative Care Consultant, Peter’s case study) and Matthew (Consultant Paediatric Neurologist, Bartholomew’s case study) recognised an expectation that professionals would be proactive in seeking relevant training and education:

‘[There are] *different channels where I can get help’* (Matthew, Consultant Paediatric Neurologist, Bartholomew’s case study).

Professionals who were inexperienced at initiating and using advance care planning reported potential issues in finding support to develop their skill levels, creating what they felt was a hierarchy between the most and least experiences professionals:

‘*It was* [a] *kind of asking colleagues thing, of who had experience of this. No, I didn’t feel there was enough help out there’* (Hannah, General Paediatrician, Miriam’s case study).

### Theme 5: Relationships

Rachel (Parent) had a good relationship with Miriam’s current consultant: many discussions between them took place over a cup of coffee in an informal setting and they had a mutual respect which promoted open and honest communication. However, Rachel described a fractious relationship with a previous consultant because she felt they had not taken time to get to know her and Miriam (Young Person):

*‘They need to know my child. I’m not a number. She’s Miriam and if you don’t know her, don’t come near us’* (Rachel, Parent, Miriam’s case study).

Gatekeeping was considered an important aspect of relationships when young people are particularly unwell, have cognitive impairment, or express strong protective tendencies towards other family members:

‘*There’s mutual protection going on here, you’ve got a young person who’s probably protecting their parents or their significant others*. . .*they’re all colluding, they’re all protecting each other. . . those are important protection mechanisms*, so *you don’t want to go in with your size nines and demolish it all*’ (Sarah, Paediatric Palliative Care Consultant, Peter’s case study).

However, building relationships prior to beginning advance care planning discussions provided opportunities to engage young people, understand familial relationships and reduce gatekeeping by parents or professionals as a barrier to engagement. Triggers to initiate advance care planning discussions were also best identified by someone who knew the family well:

‘[Advance care planning works best when] *using a combination of behavioural verbal cues and then exploring it* [sic]’ (Sarah, Paediatric Palliative Care Consultant, Peter’s case study).

Developing trusting relationships prior to initiating advance care planning was felt to allow disagreements to be discussed constructively. Such relationships meant Rachel (Parent, Miriam’s case study) felt comfortable to ask questions and was more relaxed when talking about treatments with healthcare professionals, whilst Lydia (Parent, Peter’s case study) felt more able to challenge professional judgements and decisions when Peter (Young Person) was hospitalised.

Where these relationships did not exist, some participants perceived a hierarchy in relationships in the advance care planned process. For example, Lydia (Parent) described strained relationships with professionals because of what she felt was poor communication based on hierarchies of relationships and power: she described the circumstances of Peter being in hospital and screaming in pain but the professionals appearing reluctant to listen to her concerns.

*‘I kept insisting on them doing further investigations and they wouldn’t do it. . .because they just said he’s failing, he’s failing’* (Lydia, Parent, Peter’s case study).

For Lydia, misinterpretation and miscommunication because of a focus on medical processes and diagnostic language resulted in wrong medication being administered to Peter and a misdiagnosis on at least one occasion. Similarly, the perception of a hierarchy in professional relationships in Peter’s case study was reported to be a barrier to engaging Ruth (Consultant Paediatrician). As a result, Ruth felt her power to co-ordinate support for Peter (Young Person) and Lydia (Parent) had been reduced.

‘[Co-ordinating care with different professionals who have more experience of advance care planning] *can be difficult. . .Sometimes co-ordinating things in general, not just for Peter but between hospitals, can be difficult. . .by the time we get clinic letters it’s often a few weeks after or sometimes we don’t always get them’*. (Ruth, Paediatric Consultant, Peter’s case study).

Taking time to build a trusting relationship with young people with complex needs, or who were non-verbal, was particularly important for all involved. In Peter’s case study, the relationship between Lydia (Parent) and Mary (Hospice Clinical Manager) had developed over a number of years. Both spoke fondly of each other, and expressed appreciation at the mutual input into developing their strong, open and honest relationship which facilitated the relationship and engagement of Peter:

‘*It’s taken them* [healthcare professionals] *years to get to know Peter and to understand Peter’* (Lydia, Parent, Peter’s case study).

Despite different views and experiences of relationships in the advance care planning process, the consensus within and across the case studies was that building relationships prior to initiating discussions helped foster effective communication and engage both the young people and their parents/carers.

### Theme 6: Organisational structure and culture

Most opinions (*n *= 9) about the impact of organisational structure and culture on the timing and engagement of young people in advance care planning were negative. James (Parent, Bartholomew’s case study) and Rachel (Parent, Rachel’s case study) were annoyed that communication and systems used by organisations did not allow for the seamless flow of information:

*‘You’re in and out so many times, you don’t want the same questions asking, you don’t want this, you want them to be able to press a number and all the information’s there’* (Rachel, Parent, Miriam’s case study).

An organisational culture which relied on technology created a barrier to initiating and using advance care plans because information was not easily shared within and between organisations. A more flexible and approachable person-centred culture would help move away from a process-focussed system. Similar feelings were shared by professionals, who explained that existing systems created a barrier for effective tracking and management of healthcare:

*‘A lot of children and young adults do get lost in the system in our area, in our region’* (Eve, Consultant Respiratory Physiotherapist, Bartholomew’s case study).

A rigid organisational structure providing centralised geographical locations of services, including reduced localised and out-of-hours provision, were also felt to have hindered engagement. Across the case studies, a lack of local resources resulted in having to distinguish between what services healthcare professionals would like to offer and those which could be provided:

*‘We’ve had, sometimes, nurses falling over each other, but very often that’s all nine-to-five services, and any sort of out-of-hours cover was non-existent’* (Sarah, Paediatric Palliative Care Consultant, Peter’s case study).

One professional felt the inflexible structure of organisations did not allow for 24-h care to be provided:

*‘There’s lots of challenges. . .in terms of infrastructure, co-dependency and other specialist services’* (Eve, Consultant Respiratory Physiotherapist, Bartholomew’s case study).

Barriers associated with organisational structure may be more apparent at key times in the care of young people, such as when planning for transition. Although most professionals were aware of these concerns and had begun to implement strategies to ensure transition was as smooth as possible, this was not apparent in all case studies and for some the lack of services for young adults made planning difficult. James (Parent) felt Bartholomew’s advance care plan meant approaching the transition period was a particularly troubling issue because of the uncertainty surrounding his current and future care provision:

*‘As a parent, it’s a little bit scary because we don’t know what’s happening’* (James, Parent, Bartholomew’s case study).

There was agreement from all professionals across the case studies that advance care planning is a time-consuming process and significantly impacted workload. John (Consultant Paediatric Neurologist) said he often worked evening and weekends but also recognised the flexibility provided by his organisation encouraged new ways of working:

*‘There comes an enormous responsibility and commitment and I think people need to be very clear, this is not a nine to five job* [but my organisation is] *very receptive to innovation* [and] *certainly in my experience they listen to arguments’* (John, Consultant Paediatric Neurologist, Andrew’s case study).

Overall, problems around the transition of young people to adult care, and the rigid structure of services, led to a poor experience of advance care planning for some participants. These experiences may create a barrier to the timing and implementation of advance care planning and the engagement of young people and their parents/carers in the process. Financial and workload pressures facing healthcare professionals were evident but did not affect every professional or organisation.

## Discussion

Participants shared diverse views and experiences of advance care planning for young people with the novel approach of involving the three perspectives in each case study adding to the richness of stories. Key findings relating to barriers and facilitators of engaging young people in their own care planning were apparent in the following areas: misperception of terms; hierarchies of power in relationships; and flexible and innovative organisational structure and culture.

In agreement with other research,^72,73^ the findings suggest that misperceptions of advance care planning can produce unrealistic and varied expectations and experiences of the process. For example, focussing on medical interventions, treatments and management of conditions might exclude non-medical wishes of young people. The understanding of young people was complicated by cognitive impairment rather than their conceptual understanding of advance care planning, which has been identified in previous research.^36,74^ Therefore, communication should take into consideration the maturity and cognitive ability of young people to ensure language is both age- and developmentally-appropriate. This should be supplemented with written information for the young person and family, ensuring they have a copy of the current advance care plan.

Misunderstandings of advance care planning could be attributed to younger people’s understanding of death, different cultural attitudes^12,75^ or differing levels of maturity.^76^ However, confusion with other terms, such as a carers’ assessment or end-of-life plan, suggested a lack of focus on young people in the process and a shortage of clear information being provided to both young people and their parent/carers. Open, honest, sensitive and empathetic communication was considered a clear facilitator to engaging young people and their families in advance care planning.

Relationships within, and views of, advance care planning were sometimes complicated by perceptions of hierarchies both between healthcare professionals, and between healthcare professionals and non-professionals. Developing relationships prior to initiating advance care planning appeared to reduce miscommunication, misperception of the process and decisional conflict.^77^ Perceptions of medicalised terminology and fragmented relationships contributed to a blurred understanding of advance care planning, which potentially created a barrier to engaging young people in the process.

Greater engagement in advance care planning may be developed by improving health literacy and providing opportunities for shared decision-making and joint planning.^8,78^ Perceived hierarchies may be reduced by greater access to training and education for healthcare professionals, which can improve effective communication and working relationships within advance care planning.^79^

Models of care based on the funding of services or a rigid advance care planning process reportedly produced a barrier to the engagement of young people. Conversely, an organisational structure and culture which promotes flexibility and innovation, with increased funding, opportunities for training and provision of out-of-hours care, could develop greater confidence in healthcare professionals to initiate a more person-centred and individualised process. More affordable and local or online training and education, which is available and accessible for all professionals, can ensure they are confident and able to engage young people in their care planning and reduce perceptions of hierarchies between professionals.

## Limitations and strengths

Potential limitations include the lead researcher’s lack of clinical knowledge, training or experience, which may have limited some understanding of medical conditions and processes but facilitated exploration of the topic from a naïve perspective with support from an experienced supervisory team and clinicians at lead sites. The lead researcher also attended research training as part of his PhD. Case study methodology may be considered as lacking in rigour compared to other methods of research because of the potential to distort data to match findings.^54,80,81^ However, the rigorous research design increased transparency of the study and reliability of the findings, providing greater opportunities for generalising results.^54,62^ Data analysis may have reflected personal bias but measures were taken to increase the credibility of the desired findings and rigour.^82^ Data was shared within the supervisory team and the analysis process and findings corroborated as part of the transparent study design. Consequently, idiographic generalisation of results is possible from qualitative case study research.^57,83,84^

## Conclusion

With reference to the aim and objectives of the study, a variety of views and experiences of advance care planning were expressed. Participants felt consultants should initiate the process by the time the young person is in their mid-teens, when their condition is stable, and before they transition to adult care. A range of barriers and facilitators to engagement were also identified: perceived hierarchies in relationships and potential misunderstanding of communication can lead to misperceptions of advance care planning, resulting in barriers to engaging young people and negative experiences of the care planning process. Conversely, appropriate communication, relationships developed prior to initiating advance care planning and support for everyone involved in the process, were felt to facilitate engagement. These factors were underpinned by both training and education for healthcare professionals and reported organisational structures and cultures. The broad consensus across the participants and case studies was that engaging young people in their own care planning was also felt to give them value and purpose.

Further research would be beneficial in the following areas: to ascertain how many young people with complex healthcare needs in the United Kingdom have an advance care plan; to identify and evaluate which healthcare professionals are involved in advance care planning and their training opportunities; and to develop and evaluate information and support mechanisms to facilitate the engagement of young people in advance care planning.

## Supplemental Material

sj-pdf-1-pmj-10.1177_02692163221083447 – Supplemental material for Views and experiences of young people, their parents/carers and healthcare professionals of the advance care planning process: A summary of the findings from a qualitative study Click here for additional data file. Supplemental material, sj-pdf-1-pmj-10.1177_02692163221083447 for Views and experiences of young people, their parents/carers and healthcare professionals of the advance care planning process: A summary of the findings from a qualitative study by Ben Hughes, Mary O’Brien, Anita Flynn and Katherine Knighting in Palliative Medicine
