# Supplementary material for: Views and experiences of young people, their parents/carers and healthcare professionals of the advance care planning process: A summary of the findings from a qualitative study
Source: Palliat Med. 2022 Mar 31;36(5):841–54. doi: 10.1177/02692163221083447 (PMC9087310; doi:10.1177/02692163221083447)
Supplement: sj-pdf-1-pmj-10.1177_02692163221083447 – Supplemental material for Views and experiences of young people, their parents/carers and healthcare professionals of the advance care planning process: A summary of the findings from a qualitative study [file sj-pdf-1-pmj-10.1177_02692163221083447.pdf]

<u>Interview schedule for young people</u>

1. **Please can you start by telling me a little bit about yourself. [This is an icebreaker question to start the conversation.]**

I'd like to start by asking you about your experience of having an advance care plan.

2. **What do you think an advance care plan is for?**

3. **How old were you when your Advance Care Plan was first discussed with you?**
   <u>Prompts:</u>
   - Who discussed it with you?
   - How was it raised?
   - Where did it happen?
   - Did you feel the timing was right for you? If not, when would have been better?

4. **Can you tell me about your involvement in discussions about your Advance Care Plan?**
   <u>Prompts:</u>
   - Would you have liked more/less involvement?
   - What information were you given?
   - What support were you offered?
   - Challenges to the development of the advance care plan? (If not covered above)

5. **How does having an Advance Care Plan make you feel?**
   <u>Prompts:</u>
   - Better care?
   - Communication/relationships with your family/Healthcare Professionals?
   - Challenges/benefits?
   - Enough information?
   - Who would you talk to if you have a problem?

6. **What are your thoughts on involving children in their advance care planning?**
   <u>Prompts:</u>
   - When involvement should start?
   - How it should be raised and developed?
   - Benefits?
   - Challenges?

7. **What do you think would be the best way to involve children in their advance care plan?** <u>Prompts:</u>
   - What is it?
   - Who should be involved?

- How?
- Timing?

8. **Is there anything else you would like to say about your experience of having an Advance Care Plan for yourself, or that you have thought about while we were talking?**

<u>Interview Guide for Parents/Carers</u>

[x] Indicates the child's name.

1. **Please can you start by telling me about [x], their condition and the support they currently receive?**
    a. Age
    b. Condition
    c. Current services and support received

I'd like to move on to talk specifically about your views and experience of advance care planning.

2. **Who first raised the subject of an Advance Care Plan for [x] with you?**
   <u>Prompts:</u>
    - What were the circumstances that led to the Advance Care Plan being raised/discussed?
    - Did you feel the timing was right for you? If not, when would have been better?
    - Were you given enough information about advance care plans? If not, what would have been useful?
    - Did you think they were the right person to raise the idea of the Advance Care Plan with you? If not, who do you think should have brought it up?
    - Challenges to the issue being raised? (If not covered above)

3. **What is your experience of developing an Advance Care Plan for [x] from when it was first raised to now?**
   <u>Prompts:</u>
    - Who else has been involved in the discussions? Do you think they were the right people?
    - Was paperwork readily available/easy to complete?
    - Did any circumstances/people help the process? (if not covered above)
    - Challenges to the development of the advance care plan? (If not covered above)

4. **What do you think about involving your child in the Advance Care Planning process?**
   <u>Prompts:</u>
    - Why did/didn't involve young person?
    - How?
    - When?
    - Challenges/benefits?

5. **Have you noticed any difference in your child's care since starting to use the Advance Care Plan?** <u>Prompts:</u>
    - Communication/relationships between yourselves and Healthcare Professionals, or between Healthcare Professionals from different services?

- Any aids to using an Advance Care Plan?
- Challenges in use of the Advance Care Plan??

6. **In your opinion, what is an effective Advance Care Plan for young people?**

   <u>Prompts:</u>
   - Who should be involved?
   - When?
   - How?
   - Challenges/benefits?

7. **Is there anything else you would like to say about your experience of developing and using an Advance Care Plan for your child, or any related issues?**

Interview Guide for Healthcare Professionals [x] Indicates the child's name.

1. **Please can you start by telling what your current job role is and where you work?**

   Prompts:
   - Time in current role/place of work?
   - Time working with children with life-limiting conditions or complex healthcare needs?

2. **Can you tell me about your role in the care currently provided to [x]?**

I'd like to move on to talk specifically about your views and experience of advance care planning.

3. **From your understanding, who first raised the subject of an advance care plans for [x]?**

   Prompts:
   - What were the circumstances that led to the ACPT being raised/discussed?
   - Did you feel the timing was right for the family? If not, when would have been better?
   - Did you think that [you/the person] who raised it was the right person to raise the idea of the ACP with the family? If not, who do you think should have brought it up?
   - Challenges to the issue being raised? (if not covered above)

4. **Please can you talk me through your involvement of implementing/supporting the ACP for [x] with them and their family?**

   Prompts:
   - What information was given?
   - Anyone else involved?
   - Completing the paperwork?
   - Young person involved? Expand if so. If not, why not?
   - Challenges?

5. **Do you think that you were able to provide enough information to [x]'s parents/carers (and [x] if appropriate) about the advance care plan for [x]?**

   Prompts:
   - What information is there for your professional role and/or for young people and their parents/carers?
   - Professional needs/training?
   - Do you know who to talk to/contact if you have any questions/problems?
   - If you could give any advice to someone thinking about using ACPs, what would it be?

6. **Have you noticed any differences in the care of [x] since the advance care plan was put in place?**

   Prompts:
   - Communication/Relationships between the family and Healthcare Professionals, or professionals from different services?
   - Challenges/benefits?

7. **What do you think are the key issues in terms of implementation and supporting the development of Advance Care Plans with families?**

Prompts:

- Involvement of young people in their own advance care planning discussions?
- Challenges/benefits?

8. What do you think about involving young people in the advance care planning process?

Prompts:

- Why does it happen or not happen?
- How/When are they involved?
- Challenges/benefits?

9. In your opinion, what is an effective advance care plan for young people?

Prompts:

- What is an advance care plan?
- Who should be involved?
- When?
- How?
- Challenges/benefits?

10. Is there anything else you would like to say about your experience of developing and using an ACP for [x] or that you thought about while we were talking?
